# Multiple Shoot Bud Induction and Plant Regeneration in *Madhuca indica J.F.Gmel.*: Histological, Genetic Fidelity and GC-MS Analysis

**DOI:** 10.3390/plants15121921

**Published:** 2026-06-22

**Authors:** Zishan Ahmad, Vikas Yadav, Anwar Shahzad, Anamica Upadhaya, Muthusamy Ramakrishnan

**Affiliations:** 1 State Key Laboratory of Tree Genetics and Breeding, Co-Innovation Centre for Sustainable Forestry in Southern China, Key Laboratory of National Forestry and Grassland Administration on Subtropical Forest Biodiversity Conservation, School of Life Sciences, Bamboo Research Institute, Nanjing Forestry University, Nanjing 210037, China; ahmad.bot@njfu.edu.cn (Z.A.); ramky@njfu.edu.cn (M.R.); 2 Department of Botany, Aligarh Muslim University, Aligarh 202002, India; yadavvikas535@gmail.com; 3 Botany Department, School of Life Sciences, Dr. Bhimrao Ambedkar University (Agra University), Agra 282007, India; anamica.upadhyay@gmail.com

**Keywords:** ISSR, GC-MS, *Madhuca indica*, organogenesis, RAPD

## Abstract

*Madhuca indica* J.F.Gmel. holds significant economic and industrial value due to its applications in traditional and modern medicine. Its oil is especially important for biodiesel production, owing to its high acid value and suitability as a non-edible feedstock. However, propagation is difficult due to low seed germination, seed recalcitrance, and poor rooting of stem cuttings, limiting large-scale multiplication through conventional methods. To address these limitations, a regeneration protocol using nodal explants was developed. Murashige and Skoog (MS) medium augmented with BA (5.0 µM) and NAA (0.5 µM) produced a maximum of 7.10 ± 0.11 shoots per explant with an average shoot length of 4.53 ± 0.22 cm after six weeks. Rooting was achieved on half-strength medium supplemented with IBA (1.0 µM), resulting in 4.83 ± 0.17 roots per shoot and a root length of 4.50 ± 0.20 cm. In vitro-derived plants were successfully acclimatised in Soilrite with an 82.3% survival rate. The explants were derived from aseptic seedling material, representing juvenile rather than mature elite donor sources. Direct shoot bud development was verified by histological examination. Within the resolution of the employed marker systems, no polymorphism was found utilising RAPD and ISSR markers. SEM showed similar leaf surface characteristics, and physiological and biochemical studies were carried out throughout acclimatisation. A partial overlap in metabolite composition with qualitative and relative quantitative differences between mother and in vitro-derived plants was shown by GC–MS-based profiling. Overall, the study establishes a reproducible regeneration system for *M. indica*, providing a basis for further optimisation and conservation-oriented applications.

## 1. Introduction

*Madhuca indica* J.F.Gmel. (mahua) is a medium-sized to large non-edible oil forest tree of Indian origin and a member of the Sapotaceae family and the Magnoliopsida class. It is commonly observed in various sub-tropical regions of the Indo-Pakistan subcontinent, including Bangladesh [1]. *M. indica* is economically and industrially important because of the widespread uses of its leaves, flowers, fruits, seeds, bark and timber [2,3]. It has the capacity to produce around 60 million tonnes of mahua seed oil (MO) annually [4]. MO has been widely employed in the manufacturing of biodiesel for the past few decades due to its greater acid value, which makes it appropriate for transesterification techniques [5,6]. The plant has several nutritional and medicinal values in traditional as well as in modern medicine systems. Flowers’ alcoholic and aqueous extracts demonstrate antibacterial, hepatoprotective, and analgesic properties [7,8,9]. Flower syrup is used to treat a variety of skin conditions and infections, while roasted fruits and flowers can help treat phthisis, cough, and asthma [10,11]. When taken orally, the hydroethanolic extract of its leaves exhibits hypoglycaemic action, considerably reducing blood glucose levels [12]. The sticky juice is applied to rheumatism and headaches, and the seeds are used as a laxative for constipation and piles. Seed oil is used in skin diseases, and it is generally applied as a massage oil to hydrate skin [13]. Commercially bark yields a good quantity of tannin (17%), which is used for dyeing purposes [14]. Houses, carts, and bridges are built using the tree’s robust, firm, and long-lasting heartwood [15]. Mahua seed oil, a renewable, bio-based resource, was successfully converted into hydroxyl-functional alkyd resins, which can then be used to synthesise polyurethane (PU) coatings with excellent mechanical, thermal, and anticorrosion properties [16].

Despite *M. indica*’s enormous therapeutic potential and promise to benefit society, both sexual and vegetative traditional methods of *Madhuca* propagation are plagued with numerous issues that limit their widespread growth. *M. indica* has a problem of poor seed setting and short viability, heavy rainfall in the natural habitat and seedling attack by *Loranthus* spp. in nurseries [17]. Vegetative propagation through conventional methods is also inefficient, particularly due to poor rooting performance, which restricts large-scale multiplication. While *M. indica* is the main focus of this study, *M. longifolia* var. *latifolia*, a closely related taxon with similar morphological and ecological traits, has been the subject of much prior micropropagation research in the genus *Madhuca* [18]. Since the current work does not presume synonymy between these taxa, these works are just cited for methodological comparison. Significantly, *M. indica* has unique seed oil qualities and ethnomedical qualities, which make focused propagation crucial for both industrial use and conservation.

In order to construct a repeatable regeneration process, nodal explants were obtained from aseptic seedling-derived material in the current investigation as an initial and experimentally tractable system. This offers a necessary foundation mechanism for direct organogenesis, which can then be extended to mature donor plants for real clonal multiplication and conservation-oriented applications, even though it does not reflect clonal propagation of elite mature genotypes. Reliable regeneration systems for *M. indica* are still few, necessitating the development of an effective in vitro propagation technique. The creation of such a system would facilitate the conservation of germplasm, reforestation, and the sustainable use of this understudied plant in the future. In order to create a repeatable micropropagation process, the current work solely concentrates on *M. indica* J.F.Gmel. using verified plant material. The current study used nodal explants grown from aseptic seedling material as a baseline experimental system to design an in vitro direct organogenesis strategy for *M. indica*. While SEM showed the morphological structure of regenerated leaves, histological investigation verified the direct development of shoot buds from meristematic regions. During acclimatisation, physiological and biochemical reactions were evaluated. No discernible polymorphism was found within the resolution of the marker systems employed in the genetic fidelity investigation utilising RAPD and ISSR markers. With differences in relative abundance, GC–MS-based profiling revealed qualitative overlap in phytochemical contents between mother and in vitro-derived plants. All things considered, this work creates a repeatable regeneration mechanism for *M. indica* and offers a fundamental framework for further optimisation and use.

## 2. Methodology

### 2.1. Explants, Media and Culture Condition

Nodal segment (NS) explants of *Madhuca indica* J.F.Gmel. were obtained from eight-week-old aseptic seedlings maintained in the Plant Tissue Culture Laboratory, Department of Botany, Aligarh Muslim University (AMU), Aligarh, Uttar Pradesh, India. The source plant was taxonomically identified and authenticated by Prof. M. Badruzzaman Siddiqui, Department of Botany, Aligarh Muslim University. A voucher specimen has been deposited in the Departmental Herbarium, Aligarh Muslim University, under Accession No. 24231. The donor tree was located at Aligarh, Uttar Pradesh, India (27.931255° N, 78.063402° E) (Supplementary Figure S1). Following authentication, seeds collected from the identified tree were used to establish aseptic seedlings, from which nodal segments were excised and utilised as explants for all in vitro regeneration experiments.

Following authentication, seeds collected from this single field-established, authenticated mother tree were used to establish aseptic seedlings under laboratory conditions. These seedlings were grown under controlled conditions, and uniform eight-week-old seedlings were selected for nodal segment excision and subsequently utilised as explants for all in vitro regeneration experiments.

First, the NS explants were carefully cleaned with tap water for approximately 30 min to get rid of any debris. Next, they were treated for 30 min with Bavistin™ (BASF, Mumbai, India) (carbendazim, 50% WP) at 1% (*w*/*v*), a broad-spectrum fungicide. Following a tap water wash, the NS explants were shaken continuously for approximately 15 min while being treated with 5% (*v*/*v*) Teepol™, a liquid detergent. To get rid of any remaining detergent residue, the treated NS were thoroughly cleaned once more using tap water. The last step of sterilisation was carried out under the laminar air flow cabinet wherein NS explants were submerged in 0.1% (*w*/*v*) Mercuric chloride (HgCl_2_) for about three to five minutes, followed by thorough washing (four to five times) with sterile double distilled water (DDW) to eliminate any remaining traces of HgCl_2_ before inoculation. HgCl_2_ is a highly toxic compound, and all waste solutions were handled and disposed of according to institutional hazardous chemical safety guidelines.

In all tests, NS explants were inoculated on MS basal media augmented with 0.8% (*w*/*v*) agar and 3% (*w*/*v*) sucrose with various PGRs. Every culture was cultivated in 100 cm3 Erlenmeyer flasks and 25 × 150 mm culture tubes. Once the medium’s pH was adjusted to 5.8 with 1N HCl and 1N NaOH, it was autoclaved for 15 min at 121 °C and 15 psi of pressure. All cultures were maintained at 24 ± 2 °C for 16/8 h with a PPFD of 50 µmol m^−2^ s^−1^ produced by cool white fluorescent lamps (40 W) and 55–60% relative humidity (RH).

NS explants were cultivated on varying concentrations of BA, Kn, and 2iP to produce shoot buds in order to perform direct regeneration. In order to refine the shoot regeneration potential of NS explants, the induced shoot buds were either transferred to the optimal cytokinin concentration or subcultured on the same medium. The regeneration response was then studied using different auxin combinations (IBA, IAA, and NAA) at varying concentrations. There were 20 duplicates of each explant injected at each treatment. Depending on the plant tissues’ capacity for regeneration, data for any morphogenic response or regeneration were recorded after six weeks of culture.

### 2.2. Rooting and Acclimatisation

Following removal, healthy microshoots were transferred to full- or half-strength MS media supplemented with auxin in different quantities to obtain roots. These auxins included NAA, IAA (Indole-3-acetic acid), and IBA (Indole-3-butyric acid). Whole plantlets with a fully developed shoot–root system were carefully taken out of the culture vessels and rinsed under running water to get rid of any sticking gel. After that, the plantlets were transferred to thermocol cups that were loaded with different planting substrates, including garden soil, vermicompost, and autoclaved soilrite^TM^, and kept in a culture room environment as described before. Transparent polyethylene bags with a few ventilation holes were used to enclose thermocol cups in order to verify humidity. For the first week, the cups containing the plantlets were moistened with a quarter-strength MS salt solution (which was devoid of any vitamins or sugar). This was followed by sterilised DDW and then tap water. Polyethylene bags were gradually removed once the roots had grown in order to finish the plantlets’ acclimatisation. After that, the plantlets received daily watering. Following a successful acclimatisation period, plantlets were moved to sterile earthen pots that were filled with a 2:1 mixture of garden soil and green manure. For almost a month, these potted plantlets were first maintained in a culture chamber. After that, they were collected and housed in a greenhouse for an additional month. For additional growth and development, these hardened plantlets were then transferred to real field settings.

### 2.3. Genetic Fidelity

To assess genetic fidelity, two molecular marker systems, ten RAPD primers and ten ISSR primers were employed. Nine *M. indica* plantlets that were generated in vitro and the mother plant were used for DNA extraction using the CTAB technique [19]. The quality and purity of the extracted DNA were evaluated spectrophotometrically and by agarose gel electrophoresis. A total of ten RAPD primers and ten ISSR primers were employed for PCR amplification. Each PCR reaction was performed in a final volume of 20 μL containing 2 μL of 10× PCR buffer, 0.4 μL of 10 mM dNTP mixture, 1.2 μL of 25 mM MgCl_2_, 0.2 μL Taq DNA polymerase (3 U; Thermo Scientific, Waltham, MA, USA), 2 μM primer, and approximately 40 ng template DNA. Amplifications were carried out in a thermocycler (Biometra T-Gradient Thermoblock, Göttingen, Germany). The PCR programme consisted of an initial denaturation at 94 °C for 5 min, followed by 45 cycles of denaturation at 94 °C for 1 min, annealing at 55 °C for 1 min, and extension at 72 °C for 1 min, with a final extension at 72 °C for 10 min. The amplified products were resolved by electrophoresis on 0.8% (*w*/*v*) agarose gels containing 4 μL ethidium bromide in 1× TAE buffer (pH 8.0) at 50 V for 2 h. DNA banding patterns were visualised and photographed using a UV transilluminator (Bio-Rad, Hercules, CA, USA).

### 2.4. Physiological and Biochemical Studies

For physiological and biochemical examination, vigorously growing in vitro regenerated plantlets were chosen at random. Leaf samples were collected for physiological and biochemical analyses at various acclimatisation stages, specifically 0, 7, 14, 21, and 28 days.

#### 2.4.1. Measurements of Net Photosynthetic Rate and Total Chlorophyll Concentration

Using a mortar and pestle, 100 mg of fresh tissues were extracted from the interveinal sections of the leaves and crushed in 5 mL of 80% acetone. The leaves’ total chlorophyll content was then ascertained by filtering them through Whatman’s No. 1 filter paper. The optical density (O.D.) of the filtrate was determined at wavelengths of 645 and 663 nm using a UV–visible spectrophotometer(UV-1700 Pharma Spec, Shimadzu, Kyoto, Japan). The net photosynthetic rate (P_N_) of fully developed leaves of in vitro-raised plantlets was measured using a portable infrared gas analyser at 900 µmol m^−2^ s^−1^ photosynthetically active radiation between 11:00 a.m. and 12:00 noon, on the basis of net exchange of CO_2_ between leaf and atmosphere by following the methods as mentioned in Ahmad et al. [20]. The unit of measurement for the P_N_ was µmol CO_2_ m^−2^ s^−1^.

#### 2.4.2. Biochemical Enzyme Evaluation

The antioxidant enzymes superoxide dismutase (SOD), catalase (CAT), glutathione reductase (GR), and ascorbate peroxidase (APX) were analysed by homogenising 0.2 g of fresh leaf tissue in 2.0 mL of ice-cold 0.5 M phosphate buffer (pH 7.5) containing 1% (*w*/*v*) polyvinylpyrrolidone (PVP), 1% (*v*/*v*) Triton X-100, and EDTA. The homogenate was filtered through four layers of cheesecloth and centrifuged at 15,000 rpm for 20 min at 4 °C. The resulting supernatant was used for enzyme assays. All extraction procedures were carried out at 4 °C in the dark. Soluble protein content was determined using the Bradford method, and enzyme activities were expressed on a protein basis. SOD activity was determined according to Dhindsa et al. [21] based on the inhibition of nitroblue tetrazolium (NBT) photoreduction and measured at 560 nm. CAT activity was assayed following Aebi [22] by monitoring the decomposition of H_2_O_2_ (3 mM) at 240 nm. APX activity was measured according to Nakano and Asada [23], by recording the decrease in absorbance of ascorbate (0.5 mM) at 290 nm in the presence of H_2_O_2_. GR activity was determined following Rao [24] by monitoring NADPH oxidation at 340 nm in a reaction mixture containing 0.2 mM NADPH and 0.5 mM oxidised glutathione (GSSG). Spectrophotometric measurements were performed using a UV–visible spectrophotometer. Enzyme units (EU) mg^−1^ protein were used to represent the enzymes’ activity.

### 2.5. Histological and Ultrastructural Studies

After being fixed for around 24 h in a solution of Formalin, Glacial Acetic Acid, and 70% alcohol at a ratio of 1:1:18 (*v*/*v*), the regenerating tissues at different developmental stages were preserved in 70% alcohol. The tissues were dehydrated using a succession of ethanol and xylol before being embedded in paraffin wax. A Spencer 820 rotary microtome was then used to cut the implanted tissues into longitudinal and transverse slices that were 10–12 μm thick. After that, the paraffin ribbons were applied on sanitised glass slides. The sections were cleaned with 100% alcohol after being dewaxed in a series of xylol–ethanol and stained with 1% (*w*/*v*) ethanolic safranin. To generate permanent slides, the sections were first captured using a Cannon Power Shot 640 camera and then cut under an Olympus CH20i microscope.

Leaf samples were collected for ultrastructural investigations at two distinct times: in vitro (0 days) and 6-week-old ex vitro acclimated plantlets. SEM analysis was conducted utilising the University sophisticated instrumentation facility (USIF), AMU. The materials were progressively dehydrated using a graded ethanol series after being pretreated in 5% glutaraldehyde for 24 h at room temperature. Additionally, the samples were coated with gold and dried using a CPD (critical point drying) system with carbon dioxide as the transition fluid. The coated specimens were observed using a scanning electron microscope operating at 15 kV after being affixed to aluminium stubs fixed in metallic stubs using double-sided adhesive tape. The sputter coating thickness was ~10–15 nm, the working distance was maintained at 10–12 mm, and images were captured at magnifications ranging from 500× to 5000×.

### 2.6. GC-MS Analysis

#### 2.6.1. Preparation of Plant Extract

Leaves (500 mg) collected from the field-grown and already established mother tree and in vitro-derived *Madhuca indica* J.F.Gmel. plants were used for phytochemical profiling. Fresh leaf material was thoroughly washed with running tap water followed by distilled water to remove surface contaminants and debris. The samples were air-dried, frozen in liquid nitrogen, and ground into a fine powder using a pre-chilled mortar and pestle. The powdered tissue was extracted with methanol using a Soxhlet extraction system until complete exhaustion of the plant material. The resulting extracts were concentrated and filtered through a 0.22 μm syringe filter (Genetix, India) prior to chromatographic analysis. Filtered extracts were diluted in HPLC-grade methanol to a final concentration of 2 mg mL^−1^, and 1.0 μL of each sample was injected into the GC–MS system.

#### 2.6.2. GC-MS Conditions and Compound Identification

GC–MS analysis was carried out at the Advanced Instrumentation Facility, University Science Instrumentation Centre, Jawaharlal Nehru University (JNU), New Delhi, India, using a GC–MS-QP2010 Plus system (Shimadzu, Kyoto, Japan) equipped with an RTX-5 capillary column (30 m × 0.32 mm i.d., 0.25 μm film thickness). Helium was used as the carrier gas at a constant flow rate of 3.0 mL min^−1^ with an inlet pressure of 173 kPa. The oven temperature was initially maintained at 100 °C and increased to 200 °C at a rate of 5 °C min^−1^, followed by a 6 min hold. Subsequently, the temperature was raised to 290 °C at 10 °C min^−1^ and held for an additional 10 min. The injector and ion-source temperatures were maintained at 270 °C and 250 °C, respectively. Samples were introduced using a split ratio of 1:20.

Mass spectra were recorded under electron impact (EI) ionisation at 70 eV with a scan interval of 0.5 s over a mass range of m/z 40–950. Identification of phytochemical constituents was achieved by comparing the obtained mass spectra with reference spectra available in the National Institute of Standards and Technology (NIST) and Wiley mass spectral libraries. Putative compound assignments were based on the highest spectral matching scores obtained from these databases.

### 2.7. Statistical Analysis

Twenty replicates and three repetitions were used in each experiment. SPSS ver. 17.0 (SPSS Inc., Chicago, IL, USA) was used to quantify the impact of various treatments and statistically analyse the data using a one-way ANOVA. Tukey’s test was used to assess the significance of mean variances at the 5% level of significance, and the data were expressed as mean ± standard error (SE).

## 3. Results

### 3.1. Effect of PGRs on Shoot Induction and Multiplication

**Figure 1:**
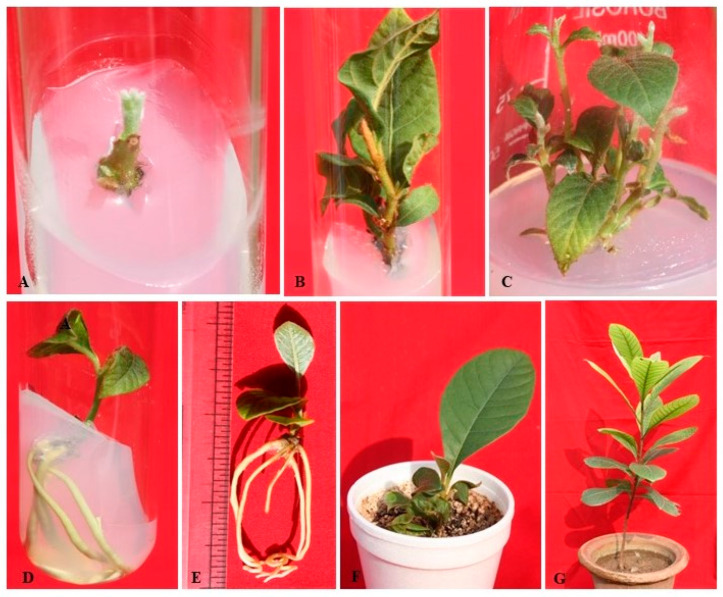
Regeneration of *M. indica* (**A**) NS cultured on MS + 5.0 µM BA (Bar = 0.41 cm); (**B**) multiple shoot initiation and regeneration on MS + 5.0 µM BA (Bar = 0.51 cm); (**C**) proliferation of shoots on MS + 5.0 µM BA + 0.5 µM NAA (Bar = 0.73 cm); (**D**) rooting on ½ MS + 1.0 µM IBA (Bar = 0.82 cm); (**E**) Exposed view of a micropropagated plant (bar = 1.0 cm). (**F**) The regenerated plantlets hardened in Soilrite (bar = 1.15 cm); (**G**) successfully acclimatised plantlets in garden soil (Bar = 5.0 cm).

Many shoot buds from NS were induced, but different types and concentrations of cytokinins tested showed varying rates of response. PGR-free MS basal medium failed to stimulate any regeneration from nodal explants (Table 1). BA was the most promising of the three cytokinins that were utilised; it caused the induction of numerous shoot buds earlier than Kn and 2-iP. The optimal concentration for the induction of a maximum of 2.86 ± 0.08 mean number of shoots of 4.50 ± 0.23 cm mean length in 60.00 ± 2.88% cultures after 6 weeks of explant inoculation was found to be 5.0 µM BA (Figure 1A,B). On 1.0 µM BA, a reduced regeneration response of 40.00 ± 2.88% was attained, resulting in an average of 2.30 ± 0.15 shoots with a length of 3.66 ± 0.12 cm. At the highest dose of 10.0 µM BA, the mean shoot length dropped to 2.46 ± 0.03 and barely reached 3.36 ± 0.08 cm in 56.66 ± 1.66% cultures. Compared to 2-iP, Kn induced a superior response to shoot regeneration. All of the evaluated parameters responded less well to any increase or decrease in the optimal concentration.

Auxin was added with optimum cytokinin (5.0 µM) to enhance the shoot regeneration capability of NS explants. A variety of auxin concentrations (0.1, 0.5, and 1.0 µM) including IBA, IAA, and NAA were added to the medium containing the optimal BA content (5.0 µM). Although BA’s combination with all three auxins gradually improved regeneration response, the synergism between BA and NAA was most effective in producing multiple shoot buds from the axillary meristem. After 6 weeks of inoculation, MS + 5.0 µM BA + 0.5 µM NAA provided the best response of all the combination treatments tested, producing an average of 7.10 ± 0.11 shoots/explant with a maximum length of 4.53 ± 0.22 cm in 81.66 ± 1.66% cultures (Figure 1C) (Table 2). Like NAA, IBA and IAA, the other two auxins, produced the best response at a 0.5 µM concentration. For multiple axillary shoot bud regeneration, the BA-IBA combination outperformed the BA-IAA synergism. Higher concentrations of these auxins led to basal callusing, which causes extensive difficulty in differentiating axillary shoot buds from NS explants.

### 3.2. Rooting and Acclimatisation

For the in vitro root induction, 4.0–5.0 cm microshoots of *M. indica* were removed from in vitro cultures and placed on agar-solidified half-strength MS medium alone or in combination with different concentrations (0.5–2.5 µM) of NAA, IAA, and IBA. Half-strength MS basal medium was used as a control. However, even six weeks after transfer, microshoots were unable to completely induce rooting on control media. However, within 12–14 days of the transfer, the addition of different auxins to the half-strength MS medium markedly increased the production of roots from the microshoots’ basal cut end. IBA was the most successful of the three auxins evaluated in promoting an early and improved rooting response. After six weeks of transfer, half-strength MS + 1.0 µM IBA produced the best rooting response of 81.66 ± 1.85% and the induction of approximately 4.83 ± 0.17 mean roots/shoot of average length 4.50 ± 0.20 cm (Table 3) (Figure 1D,E). Microshoots’ in vitro rooting response decreased when IBA concentrations were higher than 1.0 µM. At 1.0 µM, NAA likewise produced a better response, although it was less effective than IBA at promoting the in vitro rooting response. It was discovered that the in vitro root development pattern on IBA differed significantly from that on NAA and IAA. At first, the roots grown on IBA-supplemented medium were soft and light pink in colour, but as the microshoots progressed, they became thick and white in colour without any basal callusing.

After being carefully cleaned, well-established rooted microshoots were removed from the culture tubes. Out of the three potting substrates that were used, soilrite^TM^, vermicompost, and garden soil, soilrite^TM^ was the most effective and exhibited superior acclimatisation. Following a successful acclimatisation period, plantlets were moved to sterile earthen pots that were filled with a 2:1 mixture of garden soil and green manure.

**Figure 2:**
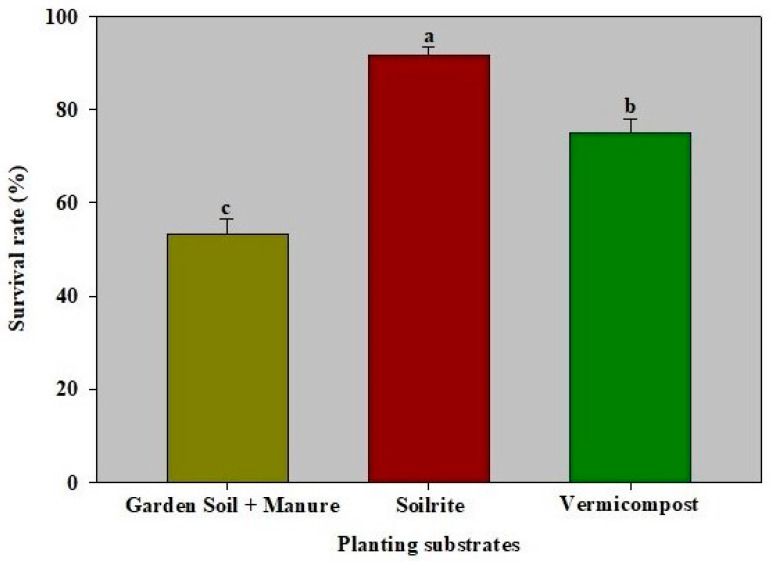
How planting material affects *M. indica* plantlets’ survival rate (%) during acclimatisation. At *p* = 0.05, bars with the same letter do not differ statistically.

For almost a month, these potted plantlets were kept in a culture chamber. After that, they were kept in a greenhouse for an additional month. Following their transfer to natural circumstances, these hardened plantlets grew healthily. Among the tested substrates, Soilrite showed the highest survival rate (82.3%), followed by vermicompost (73.6%), while garden soil + manure recorded the lowest survival (53%) (Figure 1F,G and Figure 2). The differences among treatments were statistically significant (*p* < 0.05), as confirmed by one-way ANOVA followed by post hoc analysis. The plant’s durability and lack of visible flaws were demonstrated in the field by the micropropagated plantlets, which showed the same morphological characteristics and typical growth as the mother plant.

### 3.3. Histological and Ultrastructural Studies

**Figure 3:**
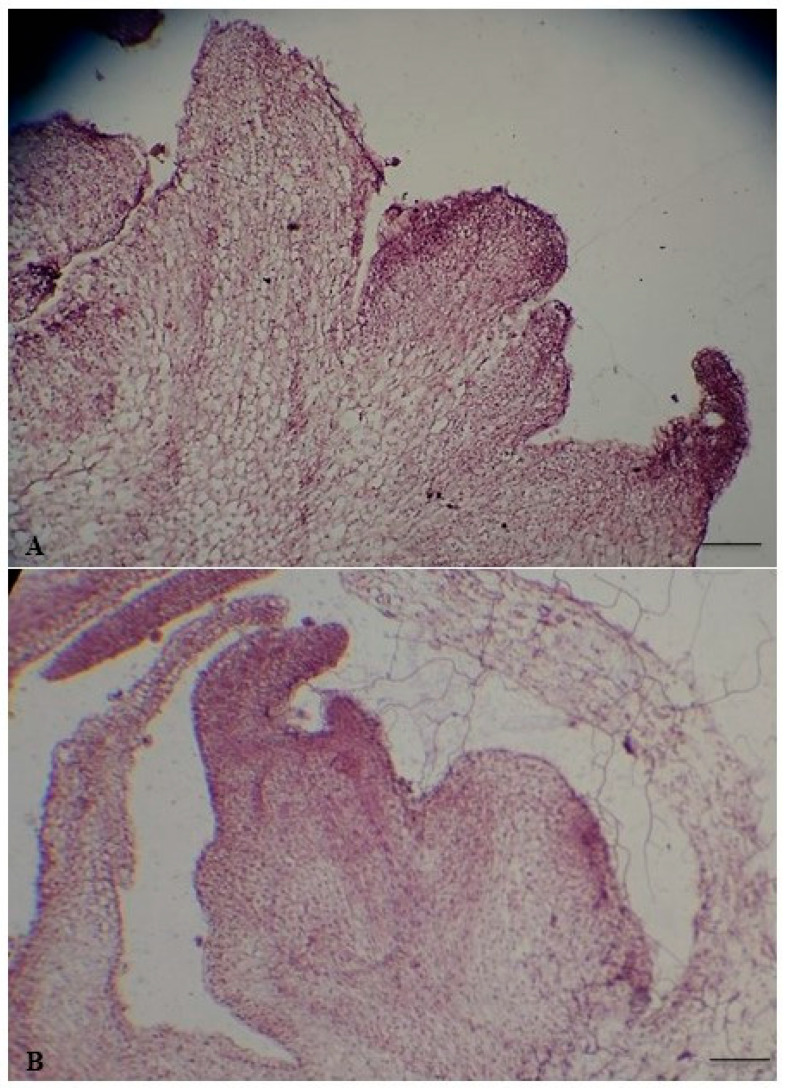
Histology of *M. indica* axillary branch proliferation via nodal explants: (**A**) Nodal explants’ vascular supply is divided into various vascular zones in their axil, and the cluster of shoot buds exhibits a well-formed apical dome and the beginning of the differentiation of leaf primordia (Bar = 160 µm). (**B**) A larger field with leaf primordia and a distinct shoot bud (Bar = 190 µm).

Histological investigation was performed by light microscopy of sectioned nodal segments fixed at distinct regenerative phases to explain in vitro organogenesis in *M. indica*. Sections from two-week-old regenerating NS produced from MS + 5.0 µM BA + 0.5 µM NAA were subjected to histological investigation, which showed the presence of a distinct meristematic zone characterised by densely stained, nucleated cells. Histological analysis confirmed that direct multiple shoot bud regeneration originated from the meristematic region of the nodal explant. Histological sections of three-week-old regenerating nodal segments showed that the apical domes that formed at the beginning of shoot buds eventually developed into complete shoot buds bordered by lateral leaf primordia and a clear vascular link with the parent tissue (Figure 3).

**Figure 4:**
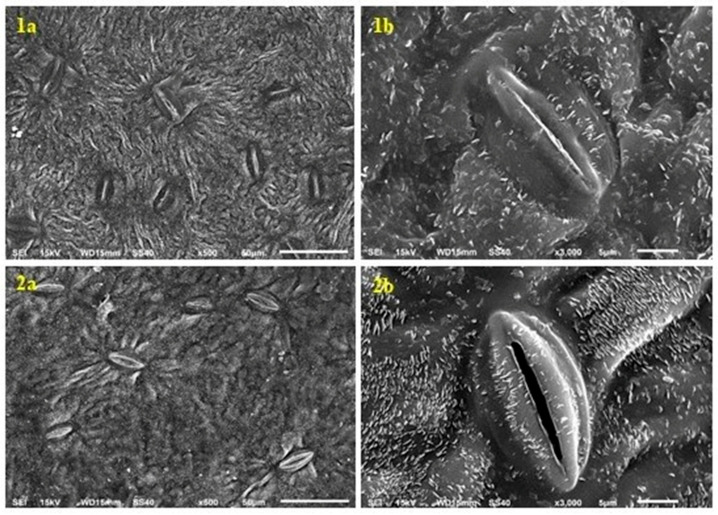
SEM surface anatomy of *M. indica* leaves grown in vitro. (**1a**) The abaxial surface of a leaf taken from an in vitro culture reveals poorly formed epidermal cells and closed stomata. (**1b**) A closer look at closed stomata on the leaf’s lower surface at 3000× reveals a lighter waxy coating on the leaf surface along with less differentiated guard cells. (**2a**) The leaf’s abaxial surface, taken from a plantlet that had been acclimated for six weeks, has fully formed open stomata and epidermal cells. (**2b**) A closer look at the stomata on the leaf’s lower surface at 3000× reveals highly waxy deposits on the leaf surface together with well-differentiated guard cells.

SEM analysis of the leaf surfaces of *M. indica* at two distinct stages, in vitro plantlets at the time of ex vitro transfer and ex vitro plantlets that were acclimated for 6 weeks, showed variations in stomatal structure, frequency, and behaviour. At these two distinct stages, variations were also seen in the quantity of trichomes and epicuticular waxy depositions on the leaf surface in addition to stomata. The irregularly formed, inflated epidermal cells with depressions and poorly developed, closed stomata that did not clearly distinguish between guard cells and stomatal apertures were visible on the abaxial surface of the leaf that was grown in vitro. There was very little epicuticular waxy accumulation on the leaf surface (Figure 4(1a,1b)). However, when the leaf surface of six-week-old ex vitro-acclimated plantlets was characterised, it revealed that the stomata were fully opened and well-developed, the epidermal cells were regular in shape, and there were numerous epicuticular waxy depositions. The stomata also had a clear aperture and distinct guard cells on the abaxial surface (Figure 4(2a,2b)).

### 3.4. Physiological and Biochemical Studies

**Figure 5:**
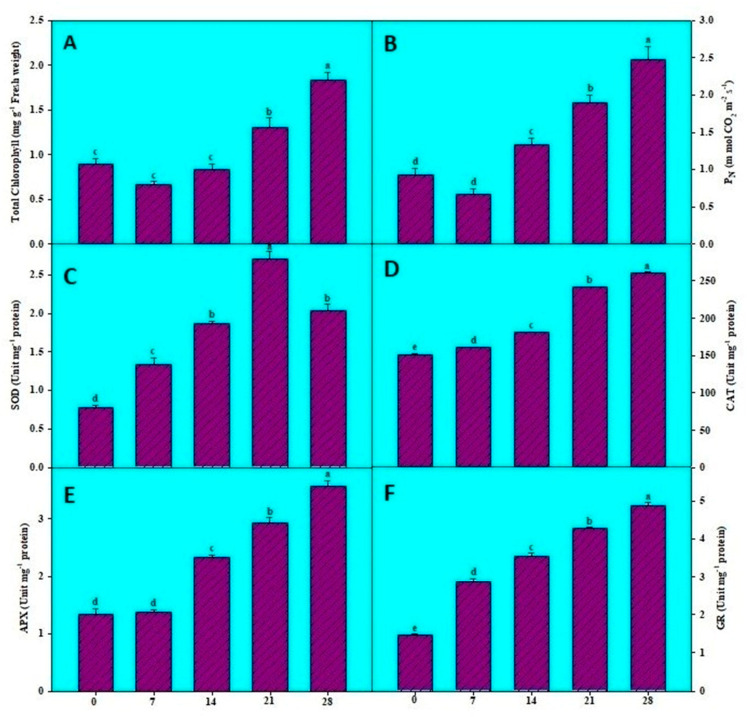
(**A**) Total chlorophyll content. (**B**) Net photosynthetic rate. (**C**) Superoxide dismutase. (**D**) Catalase. (**E**) Ascorbate peroxidase. (**F**) Glutathione reductase, during the days of acclimatisation of in vitro-derived plants of *M. indica*. Bars represent mean ± SE. Bars represented by the same letter within the response variable are not significantly different (*p* = 0.05) according to Tukey’s test at 5% probability.

Physiological studies that evaluate the net photosynthetic rate (P_N_) and total chlorophyll at various stages of ex vitro acclimatisation of in vitro-raised *M. indica* plantlets (0, 7, 14, 21, and 28 days). Chlorophyll content decreased over the first few days of acclimatisation, and a decrease of 0.95–0.69 mg g^−1^ was recorded. After that, as the acclimatisation days went by, a linear increase in the pigment content was noted. The highest amount of total chlorophyll after 28 days was 1.93 mg g^−1^, which is associated with an improvement in photosynthetic efficiency (Figure 5A).

The first week of ex vitro acclimatisation also saw an initial drop in P_N_, which was followed by a gradual increase as the acclimatisation days went by. At 0 days of acclimatisation, the P_N_ value was 0.9 µmol CO_2_ m^−2^ s^−1^; after the first week, it dropped to 0.6 µmol CO_2_ m^−2^ s^−1^. During that period, a steady increase was noted, and during 28 days of acclimatisation, a peak value of P_N_ 4.70 µmol CO_2_ m^−2^ s^−1^ was reached (Figure 5B).

As the acclimatisation process of *M. indica* plantlets grown in vitro progressed, biochemical alterations were assessed in relation to four significant antioxidant enzymes (SOD, GR, APX, and CAT). Following 0, 7, 14, 21, and 28 days of acclimatisation, SOD activity increased steadily between days 0 and 21 of acclimatisation, peaked at 2.87 Umg-1 (protein) on the 21st day of transfer, and then dropped to 1.92 Umg-1 (protein) after 28 days of acclimatisation (Figure 5C). As the acclimatisation days went on, it was discovered that CAT activity increased steadily. After 28 days of acclimatisation, it reached its highest value of 280 mmol mg^−1^ (protein) min^−1^, up from 165 mmol mg^−1^ at the beginning of the process (Figure 5D). Following 28 days of acclimatisation, the activities of both GR and APX increased linearly with the number of acclimatisation days, peaking at 4.88 mmol mg^−1^ (protein) min^−1^ and 3.84 mmol mg^−1^ (protein) min^−1^, respectively (Figure 5E,F).

### 3.5. Genetic Fidelity

**Figure 6:**
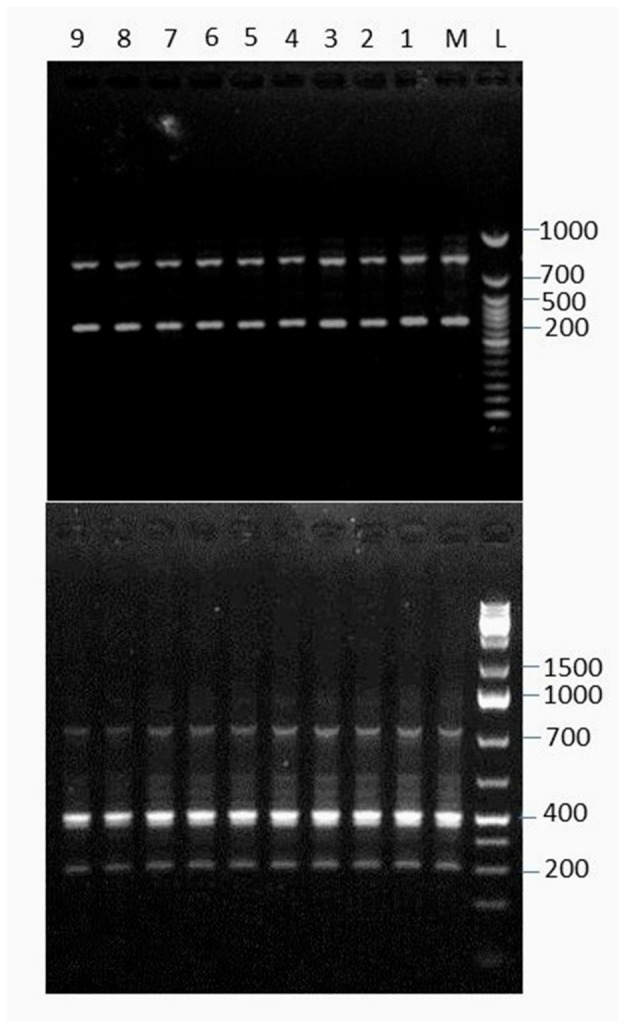
Amplified DNA profiles of the mother plant (Lane M) and in vitro plants (Lane 1–9) using the ISSR primer (UBC–827) (**bottom**) and RAPD primer (OPL–04) (**top**) demonstrate the monomorphic banding pattern. L-DNA ladder.

Two sets of PCR-based markers, RAPD and ISSR, were used to examine the genetic homogeneity of plantlets grown in vitro. Twenty distinct primers (10 RAPD and 10 ISSR) were used to examine the genetic homogeneity, which resulted in a distinct and repeatable banding pattern (Table 4 and Table 5) (Figure 6). Eight of the ten RAPD primers that were screened turned out to be responsive and produced twenty bands that could be scored with an average size range of 100–1500 bp. The number of amplified bands varied from 1 (OPL-08) to 4 (OPL-04). Nine out of ten ISSR primers that were scored yielded a total of 29 reliable and reproducible bands with an average size range of 200–2500 bp. The number of amplified bands varied from 1 (UBC-812) to 6 (UBC-827).

### 3.6. GC–MS Analysis

**Figure 7:**
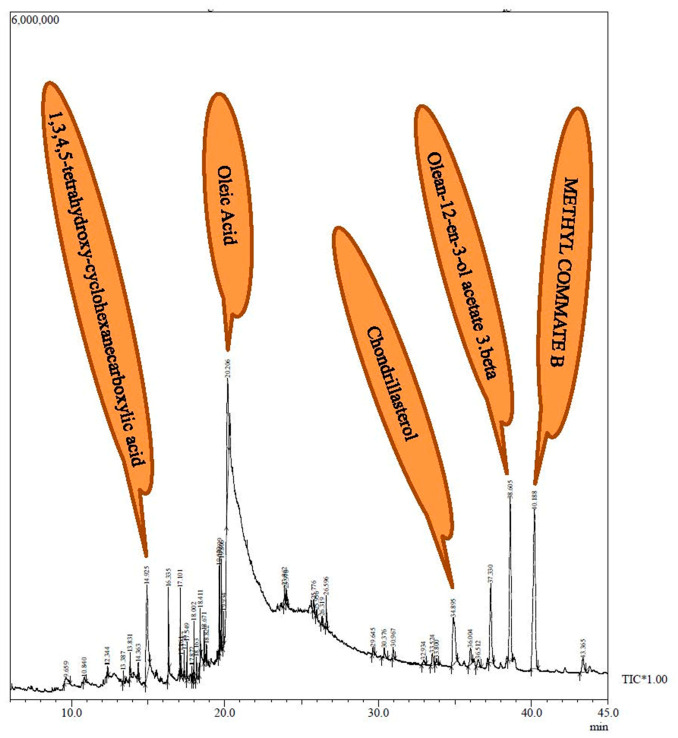
GC-MS was used to identify phytoconstituents in the methanol leaf extract of the mother plant of *M. indica*.

The presence of advantageous compounds from both sources was ascertained by GC-MS analysis of a methanolic extract prepared from the leaves of the mother *M. indica* tree and in vitro-grown plantlets. GC-MS was used to identify a diverse range of phytochemical classes detected in the mother tree’s leaf methanolic extract, including phenolic acids, ketones, aldehydes, carbohydrates, heterocyclic compounds, and hydrocarbons (Figure 7) (Table 6).

**Figure 8:**
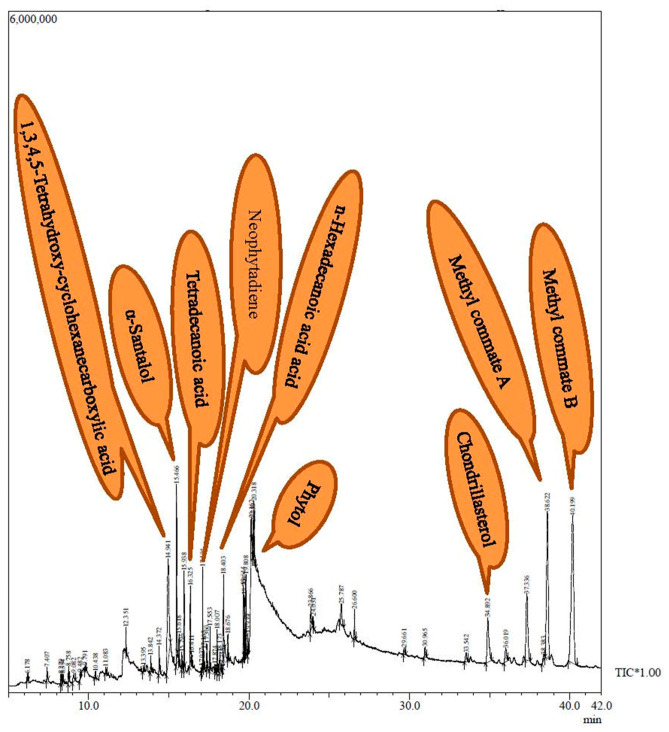
Using GC-MS, phytoconstituents were identified in the methanol leaf extract of a four-week-old *M. indica* plantlet that was cultivated in vitro.

On the other hand, GC-MS analysis of leaves taken from plantlets grown in vitro revealed a comparable number of tentative phytochemical peaks (Figure 8) (Table 7). Similar to the mother plant’s methanolic leaf extract, the leaves of micropropagated plants exhibited the presence of different key components, as represented in the table. It is acknowledged that the present GC–MS analysis is based on a single biological replicate per condition and provides a preliminary qualitative metabolite profiling. Future studies with biological replication, internal standards, and RI confirmation are required for robust quantitative comparison.

## 4. Discussion

### 4.1. Micropropagation

Medicinal plants are fundamental sources of natural products with high chemical diversity and specificity as novel lead compounds with diverse pharmacological activities [25,26,27]. Woody trees are foundational components of plant biodiversity, playing a pivotal role in maintaining ecosystem functionality by providing essential ecological services and supporting complex biological systems [28]. The unsustainable harvesting and overexploitation of valuable plant species, particularly medicinal plants, continue to deplete natural populations at an alarming rate, posing a serious threat to biodiversity and ecosystem stability in the face of growing global demand and habitat loss [29]. Thus, it is now more important than ever to protect these priceless natural resources and address the escalating ecological imbalance. A robust and cutting-edge biotechnological technology with enormous potential for the long-term preservation and mass replication of plant genetic resources is plant tissue culture [30,31,32]. For direct organogenesis in our current investigation, NS was extracted as an explant from seedlings grown in vitro. By providing vital water and nutrients needed for the growth and upkeep of developing tissues, the nutrient medium’s composition and plant growth regulators are critical in promoting the in vitro growth of explants [33,34]. A cytokinin need was identified because the nodal segment in our study failed to induce axillary bud proliferation in a control or MS medium free of any PGRs. To start the direct organogenesis process, several cytokinin concentrations—BA, Kn, and 2-iP in particular—were used. According to this investigation, the most efficient BA concentration for NS of *M. indica* shoot proliferation was 5.0 µM. Among the cytokinins, BA’s superiority was also documented in *Aflatunia ulmifolia* [35], *Casuarina equisetifolia* [36] and *Kalanchoe blossfeldiana* [37]. Fundamental variations in cytokinin perception and metabolism may be linked to BA’s better performance when compared to Kn and 2-iP. While Kn and 2-iP are isoprenoid cytokinins, BA is a member of the aromatic cytokinin group. Because aromatic cytokinins are more stable and less prone to being broken down by cytokinin oxidase/dehydrogenase (CKX) enzymes, they often show higher biological activity in tissue culture systems [38]. Furthermore, research on cytokinin receptors has revealed that benzyladenine-type cytokinins may exhibit potent receptor-binding activity, which may improve cell division stimulation and axillary meristem activation [39]. As a result, BA is frequently more successful in encouraging the production of numerous shoots in woody species and overcoming apical dominance. Higher BA concentrations (10.0 µM) may cause hormonal imbalances and physiological conditions such as hyperhydricity, which can negatively impact proper organogenic development, as seen by the decrease in shoot proliferation [40]. *Decalepisa arayalpathra* and *D. salicifolia* also showed comparable outcomes [20,41]. The optimal concentration of BA (5.0µM) was applied in combination with auxin to increase the transplant’s generating frequency. The regulation of main and lateral shoot development is brought about by the synergistic interaction of cytokinin and auxin [42,43]. As can be seen, the best combination of medium for improved growth and development was determined to be 5.0 µM of BA and 0.5 µM of NAA. In this composition, the shoot number increased from 2.86 ± 0.08 to 7.10 ± 0.11. The synergistic effect of BA and NAA has previously been established as the ideal combination in other tree species, such as *C. equisetifolia* [36]. A favourable auxin-cytokinin balance that supports axillary meristem activity while preserving cellular competence for organogenesis may be responsible for the increased shoot multiplication seen in the presence of 5.0 µM BA and 0.5 µM NAA. At the optimal treatment, the BA ratio was approximately 10:1, indicating a cytokinin-dominant hormonal environment. This finding aligns with the traditional Skoog and Miller theory, which suggests that auxin-dominant circumstances encourage root formation whereas relatively high cytokinin-to-auxin ratios favour shoot induction [44]. Recent evidence suggests that axillary meristem activity and shoot branching are regulated through complex interactions among cytokinin, auxin, and sugar signalling pathways. The improved shoot multiplication observed in the present study may therefore be attributed to the coordinated activation of these regulatory networks under the optimal BA–NAA treatment [45,46].

The type and concentration of auxins have a major impact on *M. indica* rooting. The rooting phase, particularly in tree species, is often regarded as the most critical and challenging stage of propagation [47]. Achieving high rooting efficiency is difficult due to factors such as excessive basal callus formation, accumulation of phenolic compounds, and tissue necrosis. Auxins are important for root induction and development [48]. ½ MS medium was chosen for rooting in our study, either by itself or in conjunction with the three auxins, IAA, IBA, and NAA. In our investigation, rhizogenesis was not observed in a half-strength medium devoid of any auxin, which is the control medium. A half-strength MS medium supplemented with IBA (1.0 µM) was determined to be the best rooting medium among the different auxins, leading to the formation of 4.83 ± 0.17 roots per shoot with a root length of 4.50 ± 0.20 cm. Beyond the optimum level, poor rooting was recorded and might be due to the inhibitory action of the high concentration of auxin. IBA has been found to play an important role in rooting for other plants such as in *Prunus cerasus* [49], *Hildegardia poplifolia* [50] and *Cornus sericea* [51]. In contrast to our investigation, NAA was discovered to be the most effective auxin for *C. equisetifolia* rooting [36]. Comparable responses in *Madhuca longifolia* var. *latifolia* were earlier reported by Rout and Das [17], who also found BA effective for shoot induction and IBA optimal for rooting. Our findings are consistent with their general pattern, with minor differences likely due to genotype and culture conditions.

The successful ex vitro establishment of in vitro-raised plantlets determines the excellence of the micropropagation protocol for commercial gain. By following a straightforward and traditional acclimatisation technique, the in vitro-derived plants were successfully acclimated to the field conditions. However, in order to maximise the need for the best planting substrate for each species, the impact of several planting substrates on the survival rate of acclimated plantlets was investigated. Soilrite was proven to be the most suitable of the three planting substrates used (vermicompost, garden soil + manure and Soilrite) for achieving the best survival rates. A similar kind of finding has also been observed in other studies, such as in *D. arayalpathra* [20]. However, the optimal substrate can vary significantly depending on the plant species, specific environmental conditions during acclimatisation, and the quality/composition of the various components [52,53,54]. In comparison to the mother plant, no morphological variation was observed.

### 4.2. Physiological and Biochemical Studies

The developing plantlets were grown under controlled light and temperature conditions. Additionally, plants that were transferred from in vitro to ex vitro experience physiological disablement, which raises the possibility of mortality. During this time, plants undergo significant physiological changes, which may be better understood by understanding how the photosystem and its pigments change. It is essential to look at how plantlets cultivated in vitro change physio-chemically as they move from in vitro to ex vitro growth. Net photosynthetic rate and total chlorophyll concentration in our study decreased over the initial weeks of in vitro to ex vitro transfer. Poorly developed grana and chloroplasts may cause a reduction in photosynthetic pigments, and the plants that have been directly regenerated may lose their leaves. Unexpected changes in the environment can result in photoinhibition and leaf fall in the early days [55]. The second week after acclimatisation saw the emergence of new leaves, which led to a rise in photosynthetic pigments and a subsequent gradual increase in photosynthetic rate. The photosystem’s functional health was observed across a range of light spectra due to the rise in photosynthetic pigment concentration. Similar results have also been observed for other plants, such as in *D. arayalpathra* [56] and *D. salicifolia* [41].

A crucial developmental shift that is often linked to oxidative stress is the transfer of plantlets from in vitro to ex vitro environments. Reactive oxygen species (ROS) can be produced more often when cellular redox equilibrium is upset by abrupt exposure to increased irradiance, temperature fluctuations, decreased relative humidity, and increased gas exchange [57,58,59]. Although the relative contributions of these sources were not examined in the current investigation, ROS in plants can come from a variety of cellular compartments, especially peroxisomes during photorespiratory metabolism and chloroplasts during photosynthetic electron transport [60,61]. Plants have an integrated antioxidant defence system that includes both enzymatic and non-enzymatic components to avoid oxidative damage. Following transfer to ex vitro settings, acclimatised *M. indica* plantlets in the current study showed a progressive rise in SOD, CAT, APX, and GR activities, indicating activation of antioxidant defence systems in response to oxidative stress associated with acclimatisation. By converting superoxide radicals into H_2_O_2_, which is then detoxified by CAT and APX, SOD functions as the first enzymatic line of defence. Meanwhile, GR helps regenerate reduced glutathione, which is necessary for maintaining cellular redox balance through the ascorbate–glutathione cycle [62,63]. The known ROS-scavenging mechanism that functions during environmental adaptation is compatible with the coordinated rise in these enzymes. However, the current data should be considered an indication of increased antioxidant capacity rather than conclusive evidence of particular ROS-generating pathways because ROS, subcellular ROS sources, and temporal enzyme activation kinetics were not directly examined [64,65]. Similar findings were observed in *Plantago almogravensis* [66] and *Hemidesmus indicus* [67,68].

### 4.3. Histological and SEM Studies

The purpose of the histological analysis was to examine and identify the developmental structures arising from *M. indica* regenerating explants under in vitro conditions. This analysis is crucial for understanding the origin and pathway of organogenesis. Histological investigations offer insights into the regeneration process at the microscopic level by examining cellular organisation, meristematic activity, and tissue differentiation [69,70]. This guarantees that the developmental route is accurately understood and optimised for effective plantlet production. In our results, the vascular supply of the axillary meristem was divided into distinct zones, each of which corresponded to the development of shoot buds with a clear apical dome and flanking leaf primordia. Histology of the regenerating tissue obtained on an optimised PGR concentration through nodal explants in *M. indica* confirmed the direct origin of axillary shoot buds through meristemoid formation. This study agrees with the previous findings in *Mangifera indica* [71], *Dianthus cercidifolius* [72] and *Xanthosoma sagittifolium* [69].

SEM analysis was used in this study to characterise the surface of leaves at two distinct stages, namely in vitro and six-week-old acclimatised plantlets, because it was a dependable method for observing the native superficial organisation of samples at high resolution. SEM thus provides visual evidence of physiological readiness for survival ex vitro. This aids in evaluating the success and efficiency of the acclimatisation process [73,74]. The structural adjustments made by the plants in response to the environmental change during acclimatisation are reflected in the variations in SEM analysis in *M. indica* plantlets grown in vitro and those grown ex vitro. In vitro plantlets do not need fully functional defensive features like waxy cuticles, functional stomata, or trichomes because they are produced in nutrient-rich, sterile environments with high humidity and low light levels. As a result, when transferred ex vitro, their leaves frequently exhibit uneven epidermal cells, poorly developed stomata, and low epicuticular waxes, leaving them susceptible to desiccation and environmental stress [75,76]. Plants are exposed to increased light intensity, decreased humidity, and more fluctuating air circulation and temperature as they acclimatise. The formation of trichomes, uniform epidermal cell arrangements, well-formed stomata with functional guard cells, enhanced deposition of epicuticular wax (for water retention and UV protection), and other structural modifications are all triggered by these environmental stimuli [76]. Similar findings have been observed in *Diplocyclos palmatus* and *H. populifolia* [50,77].

The histological observations confirmed the direct organogenic origin of shoot buds from nodal tissues, while SEM analysis provided qualitative evidence of progressive stomatal and epidermal differentiation during acclimatisation. However, the present study did not include quantitative measurements of histological features (e.g., meristemoid frequency or spatial distribution) or stomatal traits (e.g., density, guard cell dimensions, and aperture size), nor direct comparisons with field-grown mother plants. Therefore, the SEM observations should be interpreted as qualitative indicators of anatomical development rather than definitive evidence of complete restoration of the mother-plant phenotype. Future studies incorporating quantitative anatomical analyses and statistical comparisons among in vitro, ex vitro, and mother plants would provide a more comprehensive assessment of acclimatisation-associated structural adaptations.

### 4.4. Genetic Fidelity and GC-MS Analysis

Because somaclonal differences tend to emerge owing to in vitro stress during culture conditions, maintaining the genetic integrity of the tissue culture-generated plantlets is the most important stage in the micropropagation method, which is related to the regeneration of genetically comparable plants. Because RAPD and ISSR markers are inexpensive, reliable, easy to use, use very little DNA, and do not require radioactive probes, their usage is more obvious than that of other genetic markers. In vitro-grown plants of *M. indica* were genetically assessed using RAPD and ISSR, and the results demonstrated genetic homogeneity even after extended in vitro culture. Using RAPD and ISSR markers, it has been effectively shown that tissue culture-raised plants are genetically uniform when compared to their mother plants. These two markers have already been utilised for the genetic homogeneity assessment in several plants, including *Cymbidium aloifolium* [78], *Punica granatum* [79] and *Rheum emodi* [80].

The current study’s use of a small sample size of regenerants and a restricted set of RAPD and ISSR markers to evaluate genetic fidelity is one of its limitations. These dominant marker systems only offer a partial resolution of genome-wide variation, even though no polymorphism was found. Furthermore, the current study does not truly represent large-scale clonal multiplication of a single chosen genotype because the explants were produced from aseptic seedlings rather than elite clonal donor plants. For a thorough evaluation of genetic stability, future research using co-dominant markers or high-resolution sequencing techniques on mature donor-derived explants is advised.

The ability to document chemicals and account for complex chemical matrices in biological materials through chemical characterisation is currently recognised as a state-of-the-art technical platform. The utility and importance of GC-MS chemical profiling in plants for the identification and recording of a broad variety of phytochemicals has been shown in numerous studies [81,82,83]. In the present study, GC-MS-based metabolite profiling was employed to comparatively analyse methanolic leaf extracts from mother plants and in vitro-derived plantlets of *M. indica*. Numerous groups of phytochemicals, including phenolic compounds, aldehydes, ketones, hydrocarbons, carbohydrates, and heterocyclic components, were found in both sample types, according to the current analysis. Core metabolic pathways appear to be active in both the mother and in vitro-derived plants, based on the identification of largely comparable metabolite classes. However, these findings should not be taken as quantitative equivalency or total metabolic similarity because of the qualitative character of the investigation and the lack of biological replication and internal standards. Instead, the GC-MS results, which show discernible metabolite patterns under the particular experimental circumstances used, are presented as an initial comparative profile dataset. Differential metabolite accumulation, culture-induced physiological changes, and the analytical system’s sensitivity constraints may all have an impact on peak detection variations between samples rather than precise variations in biosynthetic capability. To enable reliable comparative and quantitative metabolomic interpretation in *M. indica*, additional research utilising biological replicates, retention index (RI) validation, and internal standard-based quantification is necessary. GC-MS has already been employed in several tree and woody plants [84,85,86].

## 5. Conclusions

Using nodal explants, this study developed an effective direct organogenesis strategy for *Madhuca indica* that resulted in high shoot multiplication, good roots, and an 82.3% acclimatisation survival rate. While SEM, physiological, and biochemical tests demonstrated gradual anatomical and physiological adaptation during acclimatisation, histological examinations verified the organogenic origin of regenerated shoots. The genetic stability of the regenerated plantlets was supported by the genetic fidelity assessment using RAPD and ISSR markers, which found no variation among the sampled regenerants within the resolution of the marker systems used. However, this conclusion is limited to the resolution of dominant marker systems and the relatively small sample size and does not represent genome-wide or long-term clonal stability assessment. GC-MS analysis identified several common metabolites in both mother plants and tissue culture-derived plants and also showed quantitative variations in the relative abundance of specific chemicals. All of these results show how useful the developed regeneration system is for *M. indica* propagation and conservation. They also lay the groundwork for future research on large-scale multiplication, long-term field assessment, and thorough metabolomic characterisation of regenerated plants under natural growing conditions. A limitation of the present study is that the regeneration protocol was developed using aseptic seedling-derived (juvenile) nodal explants and has not yet been validated on mature elite donor trees, which represent a more commercially relevant and regeneration-recalcitrant material in woody species.

## Figures and Tables

**Table 1:** Effect of different cytokinins on NS of *M. indica* for direct shoot regeneration after 6 weeks of culture.

PGR (µM)			% Response	Mean No. of Shoots/Explant	Mean Shoot Length (cm)
BA	Kn	2-iP			
Control			0.00 ± 0.00 ^h^	0.00 ± 0.00 ^g^	0.00 ± 0.00 ^f^
1.0			40.00 ± 2.88 ^efg^	2.30 ± 0.15 ^cde^	3.66 ± 0.12 ^bc^
2.5			50.00 ± 2.88 ^abcde^	2.66 ± 0.12 ^abc^	4.10 ± 0.10 ^ab^
5.0			60.00 ± 2.88 ^a^	2.86 ± 0.08 ^a^	4.50 ± 0.23 ^a^
7.5			58.33 ± 1.66 ^ab^	2.56 ± 0.03 ^abcd^	3.80 ± 0.15 ^bc^
10.0			56.66 ± 1.66 ^abc^	2.46 ± 0.03 ^bcde^	3.36 ± 0.08 ^cd^
	1.0		38.33 ± 1.66 ^fg^	2.23 ± 0.08 ^def^	2.60 ± 0.15 ^e^
	2.5		46.66 ± 1.66 ^cdef^	2.50 ± 0.11 ^abcd^	2.83 ± 0.08 ^de^
	5.0		55.00 ± 2.88 ^abc^	2.76 ± 0.09 ^ab^	3.13 ± 0.18 ^cde^
	7.5		53.33 ± 1.66 ^abcd^	2.43 ± 0.08 ^bcde^	2.76 ± 0.17 ^de^
	10.0		51.66 ± 1.66 ^abcd^	2.33 ± 0.08 ^cde^	2.60 ± 0.15 ^e^
		1.0	36.66 ± 1.66 ^g^	1.90 ± 0.05 ^f^	2.86 ±0.08 ^de^
		2.5	43.33 ± 1.66 ^defg^	2.20 ± 0.05 ^def^	2.73 ± 0.12 ^de^
		5.0	50.00 ± 2.88 ^abcde^	2.50 ± 0.05 ^abcd^	3.70 ± 0.11 ^bc^
		7.5	48.33 ± 1.66 ^bcde^	2.30 ± 0.05 ^cde^	3.36 ± 0.23 ^cd^
		10.0	46.66 ± 1.66 ^cdef^	2.06 ± 0.03 ^ef^	3.10 ± 0.20 ^cde^

Three repeated studies with 20 replicates each are represented by the mean ± SE value. According to Tukey’s test, there is no significant difference between values sharing the same letter in the column at *p* = 0.05.

**Table 2:** Effect of the optimal concentration of BA with different auxins on NS of *M. indica* for direct shoot regeneration after 6 weeks of culture.

BA (5.0 µM)		% Response	Mean No. of Shoots/Explant	Mean Shoot Length (cm)
IBA	0.1	63.33 ± 1.66 ^e^	3.73 ± 0.31 ^cd^	3.36 ± 0.21 ^bc^
	0.5	76.66 ± 1.66 ^ab^	5.83 ± 0.08 ^b^	3.76 ± 0.08 ^b^
	1.0	71.66 ± 1.66 ^bcd^	4.63 ± 0.23 ^c^	3.33 ± 0.12 ^bc^
IAA	0.1	61.66 ± 1.66 ^e^	3.33 ± 0.12 ^d^	3.00 ± 0.05 ^c^
	0.5	73.33 ± 1.66 ^bc^	3.80 ± 0.05 ^cd^	3.03 ± 0.08 ^bc^
	1.0	65.00 ± 2.88 ^de^	3.16 ± 0.08 ^d^	2.83 ± 0.03 ^c^
NAA	0.1	66.66 ± 1.66 ^cde^	3.96 ± 0.39 ^cd^	4.50 ± 0.23 ^a^
	0.5	81.66 ± 1.66 ^a^	7.10 ± 0.11 ^a^	4.53 ± 0.22 ^a^
	1.0	76.66 ± 1.66 ^ab^	5.53 ± 0.26 ^b^	4.43 ± 0.20 ^a^

Three repeated studies with 20 replicates each are represented by the mean ± SE value. According to Tukey’s test, there is no significant difference between values sharing the same letter in the column at *p* = 0.05.

**Table 3:** Impact of different auxin supplementation levels on ½ MS medium on in vitro root induction in *M. indica* following a 6-week culture period.

Treatments (µM)	% Rooting	Mean No. of Roots/Micro-Shoot	Mean Root Length (cm)	Frequency of Callogenesis
Control	00.00 ± 0.00 ^g^	00.00 ± 0.00 ^g^	00.00 ± 0.00 ^f^	−
NAA (0.5)	61.66 ± 1.66 ^def^	3.06 ± 0.08 ^cd^	3.70 ± 0.05 ^c^	−
NAA (1.0)	73.33 ± 1.66 ^b^	4.10 ± 0.05 ^b^	4.10 ± 0.08 ^b^	−
NAA (2.5)	56.33 ± 1.66 ^def^	2.80 ± 0.15 ^d^	3.33 ± 0.08 ^cd^	−
IAA (0.5)	55.00 ± 1.65 ^ef^	1.00 ± 0.05 ^f^	2.76 ± 0.14 ^e^	+
IAA (1.0)	65.00 ± 1.66 ^cd^	1.80 ± 0.05 ^e^	3.13 ± 0.14 ^de^	++
IAA (2.5)	53.33 ± 1.66 ^f^	1.20 ± 0.05 ^f^	2.60 ± 0.05 ^e^	++
IBA (0.5)	63.33 ± 1.66 ^de^	3.06 ± 0.08 ^cd^	4.15 ± 0.18 ^b^	−
IBA (1.0)	81.66 ± 1.85 ^a^	4.83 ± 0.17 ^a^	4.50 ± 0.20 ^a^	−
IBA (2.5)	71.66 ± 1.66 ^bc^	3.26 ± 0.14 ^c^	4.11 ± 0.17 ^b^	−

Three repeated studies with 20 replicates each are represented by the mean ± SE value. According to Tukey’s test, there is no significant difference between values sharing the same letter in the column at *p* = 0.05. −, +, ++ indicate no, slight, moderate, intense callusing, respectively.

**Table 4:** List of RAPD primers with their corresponding sequences and number of bands.

S. No.	Primers	Sequence (5′-3′)	No. of Bands
1.	OPL-01	GGCATGACCT	NA
2.	OPL-02	TGGGCGTCAA	3
3.	OPL-03	CCAGCAGCTT	2
4.	OPL-04	GACTGCACAC	4
5.	OPL-05	ACGCAGGCAC	2
6.	OPL-06	GAGGGAAGAG	3
7.	OPL-07	AGGCGGGAAC	2
8	OPL-08	AGCAGGTGGA	1
9	OPL-09	TGCGAGAGTC	NA
10	OPL-10	TGGGAGATGG	3

Primers showing no amplification (NA) were excluded from the calculation of scorable bands and were not considered informative for genetic fidelity analysis.

**Table 5:** List of ISSR primers with their corresponding sequences and number of bands.

S. No.	Primers	Sequence (5′-3′)	No. of Bands
1.	UBC-812	(GA)_8_A	1
2.	UBC-814	(CT)_8_A	3
3.	UBC-818	(CA)_8_G	2
4.	UBC-825	(AC)_8_T	4
5.	UBC-827	(AC)_8_G	6
6.	UBC-836	(AG)_8_YA	2
7.	UBC-848	(CA)_8_RG	5
8	UBC-855	(AC)_8_YT	NA
9	UBC-868	(GAA)_6_	2
10	UBC-880	(GGGGT)_3_G	4

Primers showing no amplification (NA) were excluded from the calculation of scorable bands and were not considered informative for genetic fidelity analysis.

**Table 6:** Phytoconstituents found in the mother *M. indica* plant’s methanol leaf extract.

Peak	Rt	Area	Area %	Name of Compound
1	9.659	674,695	1.03	1,2-benzenediol
2	10.840	241,216	0.37	Hydroquinone
3	12.344	124,369	0.19	Caryophyllene
4	13.387	214,629	0.33	Phenol, 3,5-bis(1,1-dimethylethyl)-
5	13.831	425,723	0.65	Benzene, 1,2,3-trimethoxy-5-(2-propenyl)-
6	14.363	247,802	0.38	Phthalic acid
7	14.925	6,049,768	9.19	1,3,4,5-tetrahydroxy-cyclohexanecarboxylic acid
8	16.335	1,535,298	2.33	Tetradecanoic acid
9	17.101	1,093,922	1.66	Neophytadiene
10	17.161	229,794	0.35	1-Dodecanol, 3,7,11-trimethyl-
11	17.357	416,865	0.63	Z,E-2,13-Octadecadien-1-ol
12	17.549	490,024	0.74	Neophytadiene
13	18.002	829,062	1.26	Hexadecanoic acid, methyl ester
14	18.411	1,059,939	1.61	n-Hexadecanoic acid
15	18.671	421,769	0.64	Ethyl pentadecanoate
16	18.822	281,909	0.43	4,4-Ethylenedioxy-1-pentylamine
17	19.639	1,152,127	1.75	9,12-Octadecadienoic acid, methyl ester
18	19.699	1,264,323	1.92	9-Octadecenoic acid (Z)-, methyl ester
19	19.806	1,259,470	1.91	Phytol
20	19.934	479,127	0.73	Methyl stearate
21	20.206	5,918,082	8.99	Oleic Acid
22	23.862	554,828	0.84	Palmitin, 2-mono-
23	23.976	452,717	0.69	Docosanoic acid, methyl ester
24	25.956	148,433	0.23	Spathulenol
25	26.319	309,020	0.47	cis-11-Eicosenamide
26	26.596	709,950	1.08	Squalene
27	29.645	277,372	0.42	gamma.-Tocopherol
28	30.376	455,318	0.69	STIGMAST-5-EN-3-OL, (3.BETA.)-
29	30.967	411,298	0.62	Vitamin E
30	32.934	244,424	0.37	5-(7a-Isopropenyl-4,5-dimethyl-octahydro-inden-4-yl)-3-methyl-penta-2,4-dien-1-ol
31	33.524	624,105	0.95	Stigmasterol
32	33.800	256,423	0.39	1,1,6-trimethyl-3-methylene-2-(3,6,9,13-tetramethyl-6-ethenye-10,14-dimethylene-pentadec-4-enyl)cyclohexane
33	34.895	4,717,260	7.17	Chondrillasterol
34	36.004	906,166	1.38	METHYL COMMATE A
35	36.512	494,512	0.75	Cholest-7-en-3-ol, 2,2-dimethyl-, (3.beta.,5.alpha.)-
36	37.330	4,883,761	7.42	METHYL COMMATE B
37	38.605	10,040,884	15.26	Olean-12-en-3-ol, acetate, (3.beta.)-
38	43.365	1,102,199	1.67	Kolavelool

**Table 7:** Methanol leaf extract of the in vitro-propagated *M. indica* plant contained phytoconstituents.

Peak	Rt	Area	Area %	Name of Compound
1	6.178	104,268	0.17	Cyclohexene, 1-methyl-4-(1-methylethenyl)
2	7.407	259,597	0.42	1,6-octadien-3-ol, 3,7-dimethyl-
3	8.279	204,822	0.33	6-octenal, 3,7-dimethyl
4	8.394	137,456	0.22	Cyclohexanone, 5-methyl-2-(1-methylethyl)-, cis-
5	8.758	244,429	0.39	Cyclohexanol, 5-methyl-2-(1-methylethyl)-,
6	9.082	360,642	0.58	BENZENE, 1-METHOXY-4-(2-PROPENYL)-
7	9.482	158,120	0.25	6-OCTEN-1-OL, 3,7-DIMETHYL-
8	9.791	58,355	0.09	1,2-Dihydropyridine, 1-(1-oxobutyl)-
9	10.438	133,963	0.21	4-hexen-1-ol, 2-isopropenyl-5-methyl-, acetate
10	11.083	157,846	0.25	Triacetin
11	12.351	328,277	0.53	4,11,11-Trimethyl-8-methylenebicyclo[7.2.0]undec-3-ene
12	13.395	94,198	0.15	Phenol, 3,5-bis(1,1-dimethylethyl)-
13	13.842	307,549	0.49	Benzene, 1,2,3-trimethoxy-5-(2-propenyl)-
14	14.372	693,989	1.11	1,2-benzenedicarboxylic acid, diethyl ester
15	14.941	6,307,922	10.10	1,3,4,5-Tetrahydroxy-cyclohexanecarboxylic acid
16	15.466	2,776,379	4.45	α-Santalol
17	15.618	466,257	0.75	Bergamotol, Z-.alpha.-trans-
18	15.796	378,548	0.61	(Z)-epi-.beta.-Santalol
19	15.938	1,476,010	2.36	β-Santalol
20	16.325	1,363,237	2.18	Tetradecanoic acid
21	16.411	141,076	0.23	Lanceol, cis
22	17.037	79,799	0.13	(2e)-3,7,11,15-Tetramethyl-2-hexadecene
23	17.104	1,333,227	2.13	Neophytadiene
24	17.165	266,692	0.43	1-Dodecanol, 3,7,11-trimethyl-
25	17.360	452,361	0.72	7,11,15-Trimethyl-3-methylenehexadec-1-ene
26	17.553	604,923	0.97	Neophytadiene
27	18.007	637,678	1.02	Hexadecanoic acid, methyl ester
28	18.403	1,587,147	2.54	n-Hexadecanoic acid
29	18.676	386,126	0.62	Hexadecanoic acid, ethyl ester
30	19.644	965,160	1.55	9,12-Octadecadienoic acid, methyl ester
31	19.704	779,679	1.25	(9e,12e)-9,12-Octadecadienoyl chloride
32	19.757	48,459	0.08	9-Octadecenoic acid (z)-, methyl ester
33	19.808	1,267,274	2.03	Phytol
34	19.939	291,869	0.47	Methyl stearate
35	20.162	344,291	0.55	Oleic acid
36	20.259	471,303	0.75	Trans,trans-9,12-Octadecadienoic acid, propyl ester
37	20.318	1,130,944	1.81	9-Octadecenoic acid (Z)
38	23.866	700,289	1.12	Hexadecanoic acid, 2-hydroxy-1-(hydroxymethyl)ethyl ester
39	24.031	163,016	0.26	1,2-Benzenedicarboxylic acid
40	25.787	961,193	1.54	Lup-20(29)-ene-3,28-diol, (3.beta.)-
41	26.600	567,684	0.91	Squalene
42	29.661	250,651	0.40	γ-Tocopherol
43	30.965	427,051	0.68	Vitamin E
44	33.542	470,297	0.75	Stigmasterol
45	34.892	3,219,169	5.15	Chondrillasterol
46	36.019	827,053	1.32	Methyl commate A
47	37.336	4,410,689	7.06	Methyl commate B
48	38.383	267,266	0.43	1-Isopropenyl-4,5-dimethylbicyclo[4.3.0]nonan-5-ylmethyl phenyl sulfoxide

## Data Availability

Protein sequences, DNA/RNA sequences, and other data types that need to be deposited in public repositories are not included in the datasets created and/or examined in this study. Our research focuses on tissue culture experiments, and the publication contains all pertinent experimental procedures and findings. The authors certify that all experimental studies on *Madhuca indica*, including the gathering of plant material, were carried out in compliance with the rules established by the department of the university.

## Supplementary Materials

The following supporting information can be downloaded at: https://www.mdpi.com/article/10.3390/plants15121921/s1, Figure S1: The donor tree *Madhuca indica* J.F.Gmel.
