# Supplementary figures and images for: Multiple Shoot Bud Induction and Plant Regeneration in Madhuca indica J.F.Gmel.: Histological, Genetic Fidelity and GC-MS Analysis
Source: Plants (Basel). 2026 Jun 22;15(12):1921. doi: 10.3390/plants15121921 (PMC13307094; doi:10.3390/plants15121921)

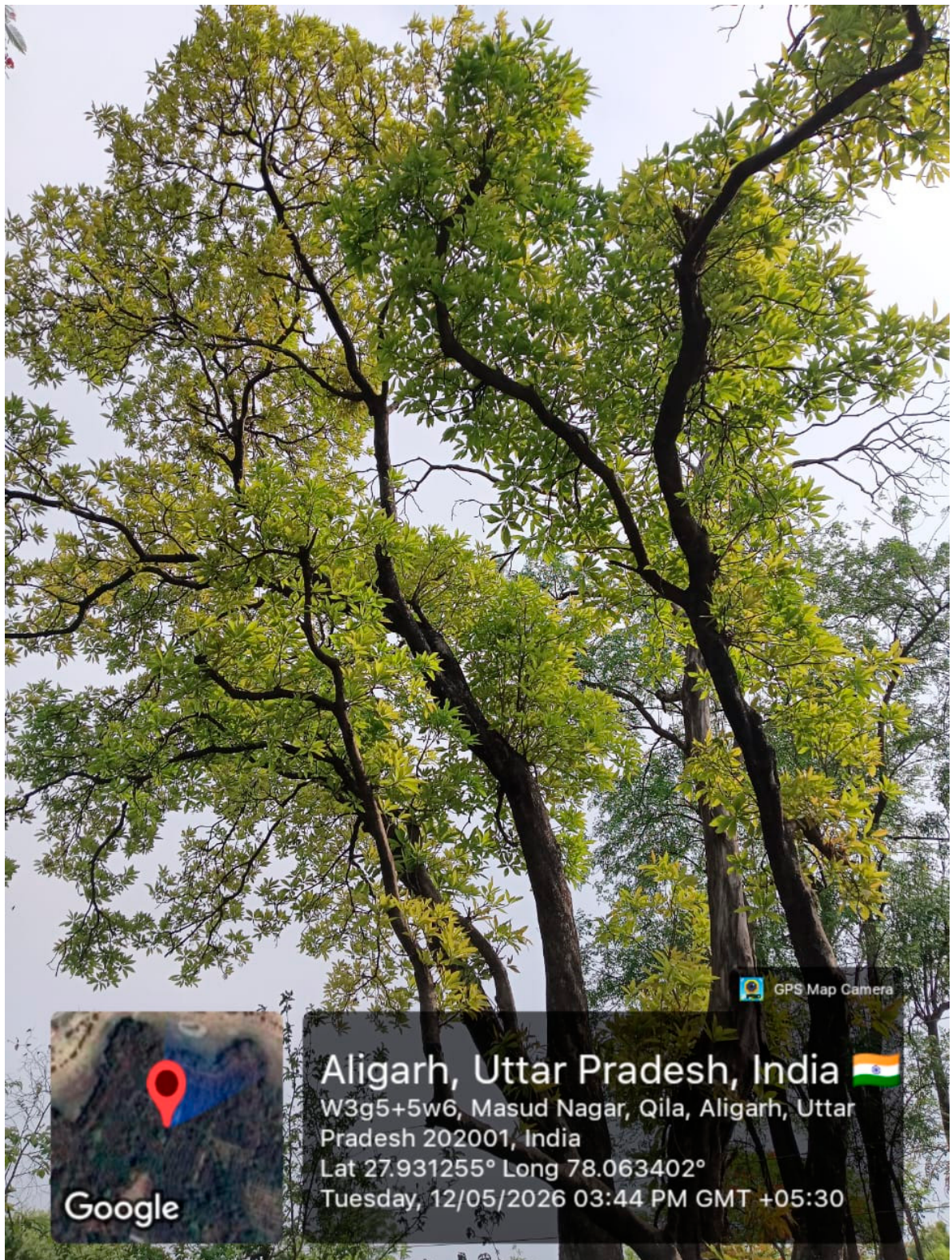

Figure S1: The donor tree *Madhuca indica* J.F.Gmel.

Supplement: Supplementary file 1 [file plants-15-01921-s001.zip › plants-4306657-supplementary.pdf]
